# Repetitive Glucose Spikes Accelerate Atherosclerotic Lesion Formation in C57BL/6 Mice

**DOI:** 10.1371/journal.pone.0136840

**Published:** 2015-08-27

**Authors:** Yuki Shuto, Akira Asai, Mototsugu Nagao, Hitoshi Sugihara, Shinichi Oikawa

**Affiliations:** 1 Department of Endocrinology, Diabetes and Metabolism, Graduate School of Medicine, Nippon Medical School, Tokyo, Japan; 2 Food and Health Science Research Unit, Graduate School of Agricultural Science, Tohoku University, Sendai, Japan; Showa University School of Pharmacy, JAPAN

## Abstract

**Background:**

A number of epidemiological studies demonstrated that postprandial hyperglycemia is a risk factor for cardiovascular disease in individuals with impaired glucose tolerance. Although several laboratory studies have addressed the plausible causal role of postprandial acute hyperglycemia (glucose spikes) in the development of atherosclerosis, there is little convincing evidence *in vivo* whether the atherosclerotic lesion formation can be accelerated solely by glucose spikes. Here, we assessed the effect of repetitive glucose spikes on atherosclerotic lesion formation in mice.

**Methods:**

Female C57BL/6 mice were fed an atherogenic diet from 8 to 28 weeks of age. During the atherogenic diet feeding period, the mice orally received a glucose solution (50 mg glucose/mouse; G group) or water (W group) twice daily, 6 days a week. Atherosclerotic lesion formation in the aortic sinus was quantitatively analyzed in serial cross-sections by oil red O staining.

**Results:**

G group mice showed transient increases in blood glucose level (~5 mmol/L above W group), and the levels returned to levels similar to those in W group mice within 60 min. No significant differences in glucose tolerance, insulin sensitivity, and plasma lipid profiles were observed after the 20-week repetitive administration between the 2 groups. G group mice showed an approximately 4-fold greater atherosclerotic lesion size in the aortic sinus than W group mice. Gene expression levels of *Cd68* and *Icam1* in the thoracic aorta were higher in G group mice than in W group mice.

**Conclusions:**

These results indicate that glucose spikes can accelerate atherosclerotic lesion formation, with little influence on other metabolic disorders. Repetitive glucose administration in wild-type mice may serve as a simple and useful approach to better understanding the causal role of glycemic spikes in the development of atherosclerosis.

## Background

An increasing body of epidemiological evidence links diabetes to atherosclerotic cardiovascular disease [1]. Even in individuals with impaired glucose tolerance (IGT), which generally precedes the clinical onset of type 2 diabetes, postprandial hyperglycemia has been reported to be associated with an increased risk of cardiovascular disease [2,3]. Moreover, several epidemiological studies indicate that post-glucose challenge hyperglycemia is a better predictor of cardiovascular events and all-cause mortality than fasting blood glucose level [4–7].

Since repetitive postprandial acute hyperglycemia (glucose spikes) is considered to result in an increase in cardiovascular events in individuals with IGT, several laboratory studies have addressed the plausible underlying mechanisms [2,3,8]. However, largely due to the lack of suitable animal models that well explain the pathophysiological conditions of IGT, the causal mechanisms underlying IGT-induced atherosclerosis remain to be fully elucidated *in vivo*.

Recently, we established 2 mouse lines, with distinctively different susceptibilities to diet-induced glucose intolerance, by selective breeding (designated *s*electively bred *d*iet-induced *g*lucose intolerance-*p*rone [SDG-P] and-*r*esistant [SDG-R]) [9,10], and demonstrated that SDG-P mice showed accelerated atherosclerotic lesion formation compared to SDG-R mice [11]. Since SDG-P mice manifested post-glucose challenge hyperglycemia without overt fasting hyperglycemia, the mice may serve as useful animal models for studying IGT-related disorders. However, SDG-P mice also showed greater body weight gain and moderate insulin resistance compared to SDG-R mice. Thus, it is difficult to discriminate the effect of glucose spikes *per se* from those of concomitant metabolic factors on the atherosclerotic process in these mice. In this study, we assessed atherosclerotic lesion formation in atherogenic diet-fed wild-type C57BL/6 mice that received repetitive glucose administration, to focus on the effect of glycemic spikes on the pathogenesis of atherosclerosis.

## Methods

### Ethics statement

This study was conducted with the approval from the institutional animal care and use committee of Nippon Medical School (25–081 and 26–128) and in strict accordance with the committee’s animal care guidelines. All efforts were made to minimize suffering and the number of animals used. No obvious adverse events were observed.

### Animals

Specific pathogen-free 7-week-old female C57BL/6J mice were purchased from CLEA Japan (Tokyo, Japan). Female mice are known to be more susceptible to atherosclerotic lesion formation than males [12,13]. After acclimatized with a standard rodent chow (MF; Oriental Yeast, Tokyo, Japan) for 1 week, the mice (17.7 ± 0.2 g, n = 16) were assigned to 2 groups, *i*.*e*., glucose-administered (G, n = 8) and water-administered (W, n = 8), ensuring equal weight average. The G group mice received a 20% glucose solution (50 mg glucose/mouse) by oral gavage twice a day (08:00 and 16:00), 6 days a week for 20 weeks. The W group mice similarly received distilled water alone. During the 20-week administration period, both groups of mice were fed an atherogenic diet (F2HFD1, Oriental Yeast) based on Paigen’s formulation [14]. The mice were housed in standard plastic cages (235 × 325 × 170 [h] mm; 4 mice/cage) with paper chip bedding and maintained in a temperature controlled room (22–24°C) with a 14-h light (06:00–20:00 h)/10-h dark cycle, with free access to food and water. The cages, bedding, food, and water bottles were changed weekly.

### Diurnal blood glucose profile

Diurnal blood glucose levels were measured in the 2nd, 11th, and 20th week of the administration period. Blood samples were obtained by tail bleeding at 20 min before and at 20, 60, and 180 min after the administration of glucose (in G group) or water (in W group). Blood glucose levels were measured with a glucose sensor (Glutest Neo Super; Sanwa Kagaku Kenkyusho, Nagoya, Japan).

### Oral glucose tolerance test (OGTT)

Glucose tolerance was evaluated by OGTT in the 19th week of the administration period. After an overnight fast, both groups of mice were given a 20% glucose solution (50 mg glucose/mouse) by oral gavage. Blood glucose levels were measured before and at 15, 30, 60, and 120 min after glucose administration as described above.

### Insulin tolerance test (ITT)

ITT was performed in the 20th week of the administration period. After 6 h-fasted blood glucose levels were measured, insulin (Humulin R, Eli Lilly Japan, Tokyo, Japan) was intraperitoneally injected at 0.2 U/kg of body weight. Blood glucose levels at 15, 30, 60, and 90 min after the injection were measured as described above.

### Plasma lipids and insulin

At the end of the administration period, blood was collected 2–3 h after the final administration of glucose (G group) or water (W group) from the inferior vena cava under deep anesthesia with intraperitoneal sodium pentobarbital injection (100 mg/kg). Total cholesterol, high-density lipoprotein (HDL) cholesterol (sodium phosphotungstate-magnesium chloride precipitation method), triacylglycerol, and non-esterified fatty acid levels in the blood plasma were measured using commercial kits (Wako Pure Chemical, Osaka, Japan). Non-HDL cholesterol level was calculated from the total and HDL cholesterol levels. Plasma insulin concentration was measured with an Ultra Sensitive Mouse Insulin ELISA (Morinaga Institute of Biological Science, Yokohama, Japan).

### Evaluation of atherosclerosis in aortic sinus

Atherosclerotic lesion size in the aortic sinus was quantitatively analyzed based on the method of Paigen *et al*. [13] with modifications [11]. In brief, after perfusion in situ with saline, the heart was isolated and fixed with 4% formaldehyde in phosphate buffered saline. It was then immersed in 30% sucrose solution and cut at a plane parallel to the atrial appendages. The upper part, including the aortic root, was embedded in optimum cutting temperature compound (Sakura Finetek, Tokyo, Japan). Cryostat sections were cut from the left ventricular outflow tract until 3 valve cusps were exposed. Thereafter, 10-μm-thick 45 serial cross-sections were prepared. Of the serial sections, every 5 sections (9 sections, each separated by 50 μm) were stained with oil red O, and then counterstained with hematoxylin. The oil red O-stained area was determined manually from the photomicrograph images by an observer who was not aware of the group allocation (YS), using Photoshop Elements software (Adobe Systems, San Jose, CA). The oil red O-stained area of the 9 sections was averaged and expressed as the mean lesion size for each mouse. Immunohistochemical staining was also performed to confirm macrophage infiltration into the lesions. In brief, adjacent sections to the oil red O-stained ones were stained with MOMA-2 rat monoclonal antibody to mouse macrophages (AbD Serotec, Oxford, UK) using Vectastain Elite ABC kit (Vector, Burlingame, CA), followed by hematoxylin staining.

### Gene expression in thoracic aorta

Total RNA was extracted from the thoracic aorta using Isogen II reagent (Nippon Gene, Tokyo, Japan), and the cDNA was generated using SuperScript VILO (Life Technologies, Carlsbad, CA). Quantitative polymerase chain reaction (PCR) was performed using TaqMan Gene Expression Assays (Life Technologies), and the differential expression was determined by the 2^-ΔΔCt^ method, with *Actb* (β-actin) used as the internal control.

### Statistical analysis

All data are expressed as mean ± standard error of the mean (SEM). Values of *p* < 0.05 by Student *t*-test were considered statistically different between G and W groups.

## Results

### Blood glucose fluctuation

**Fig 1:**
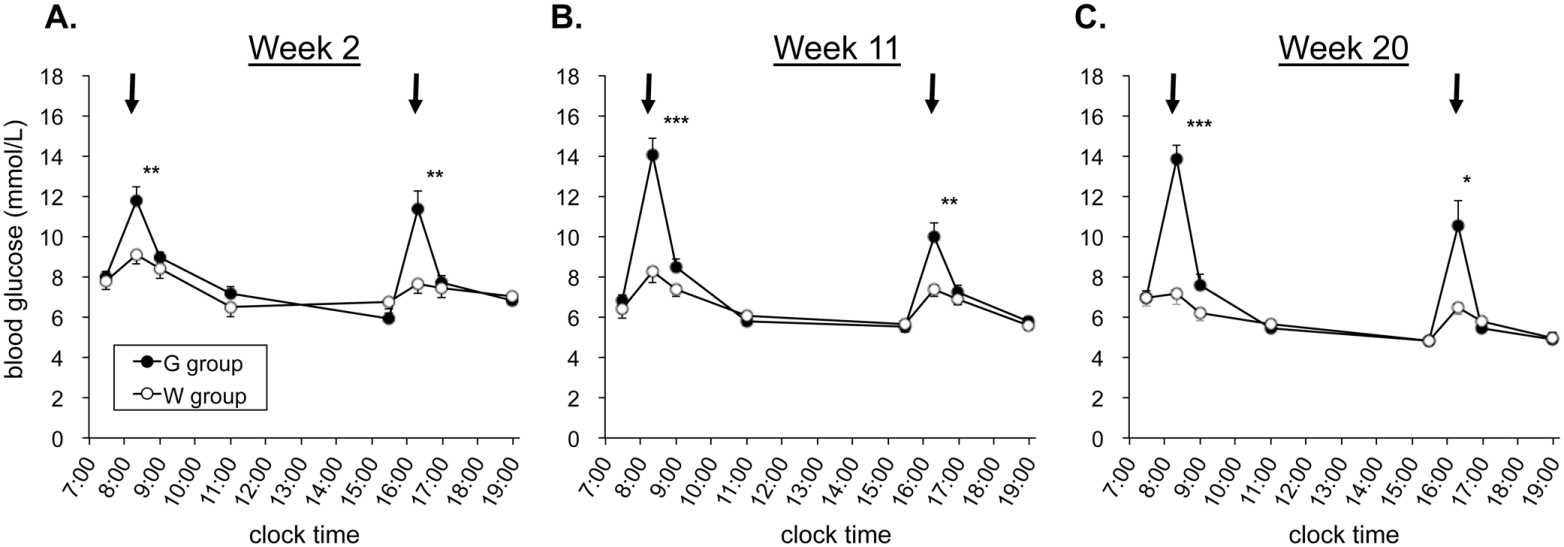
Diurnal blood glucose profile. Blood glucose levels were monitored in the 2nd (A), 11th (B), and 20th (C) week of repetitive glucose (G group) or water (W group) administration. Arrows indicate the administration time (08:00 and 16:00). Values are expressed as mean ± SEM of 8 mice for each group. *p < 0.05, **p < 0.01, ***p < 0.001 *vs*. W group.

Fig 1 shows diurnal blood glucose profiles in the 2nd, 11th, and 20th week of the administration period. G group mice showed transient increases in blood glucose level 20 min after each administration, by ~5 mmol/L above that in W group. The blood glucose levels in G group mice returned to levels similar to those in W group within 60 min.

### Glucose tolerance and insulin sensitivity

**Fig 2:**
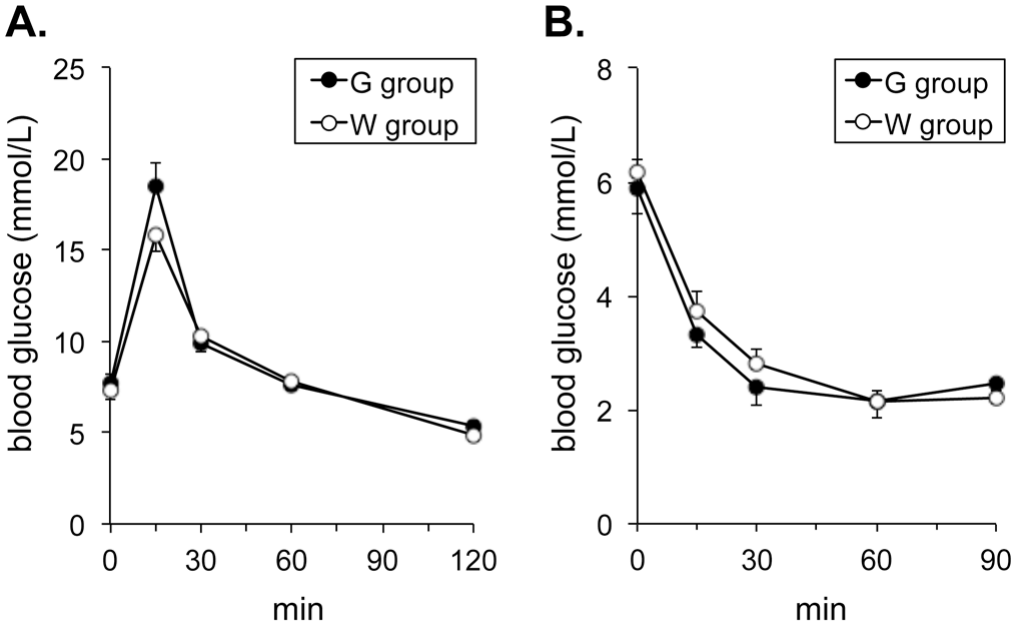
Blood glucose levels in oral glucose tolerance test (A) and insulin tolerance test (B). Values are expressed as mean ± SEM of 5 mice for each group. No significant differences were seen in blood glucose levels between G and W groups at any time point in the oral glucose tolerance test and the insulin tolerance test.

There were no significant differences in blood glucose levels in the OGTT between G and W groups at any time point (Fig 2A). The ITT showed no significant difference in insulin sensitivity between the 2 groups (Fig 2B).

### Body weight, tissue weight, plasma lipids, and insulin

There were no significant differences in body weight gain, liver weight, and gonadal fat mass between the 2 groups after the 20-week administration period (Table 1). No significant differences were seen in plasma insulin, total cholesterol, HDL cholesterol, non-HDL cholesterol, and triacylglycerol levels after the administration period (Table 1).

**Table 1:** Body weight gain, relative tissue weight, plasma insulin, and plasma lipid profile after the 20-week administration period.

	G group	W group	*p* value
**Body weight gain (g/20 weeks)**	4.3 ± 0.4	3.5 ± 0.4	0.13
**Tissue weight (mg/g body weight)**			
** Liver**	89.6 ± 2.5	86.1 ± 3.0	0.38
** Gonadal fat**	11.5 ± 0.7	12.4 ± 0.7	0.36
**Insulin (ng/mL)**	0.41 ± 0.05	0.32 ± 0.02	0.15
**Plasma lipid (mmol/L)**			
** Total cholesterol**	5.44 ± 0.50	5.19 ± 0.28	0.67
** HDL cholesterol**	0.36 ± 0.04	0.48 ± 0.06	0.13
** Non-HDL cholesterol**	5.08 ± 0.50	4.71 ± 0.29	0.53
** Triacylglycerols**	0.17 ± 0.02	0.16 ± 0.02	0.64

Values are expressed as mean ± SEM of 8 mice for each group.

### Atherosclerotic lesion formation in aortic sinus

**Fig 3:**
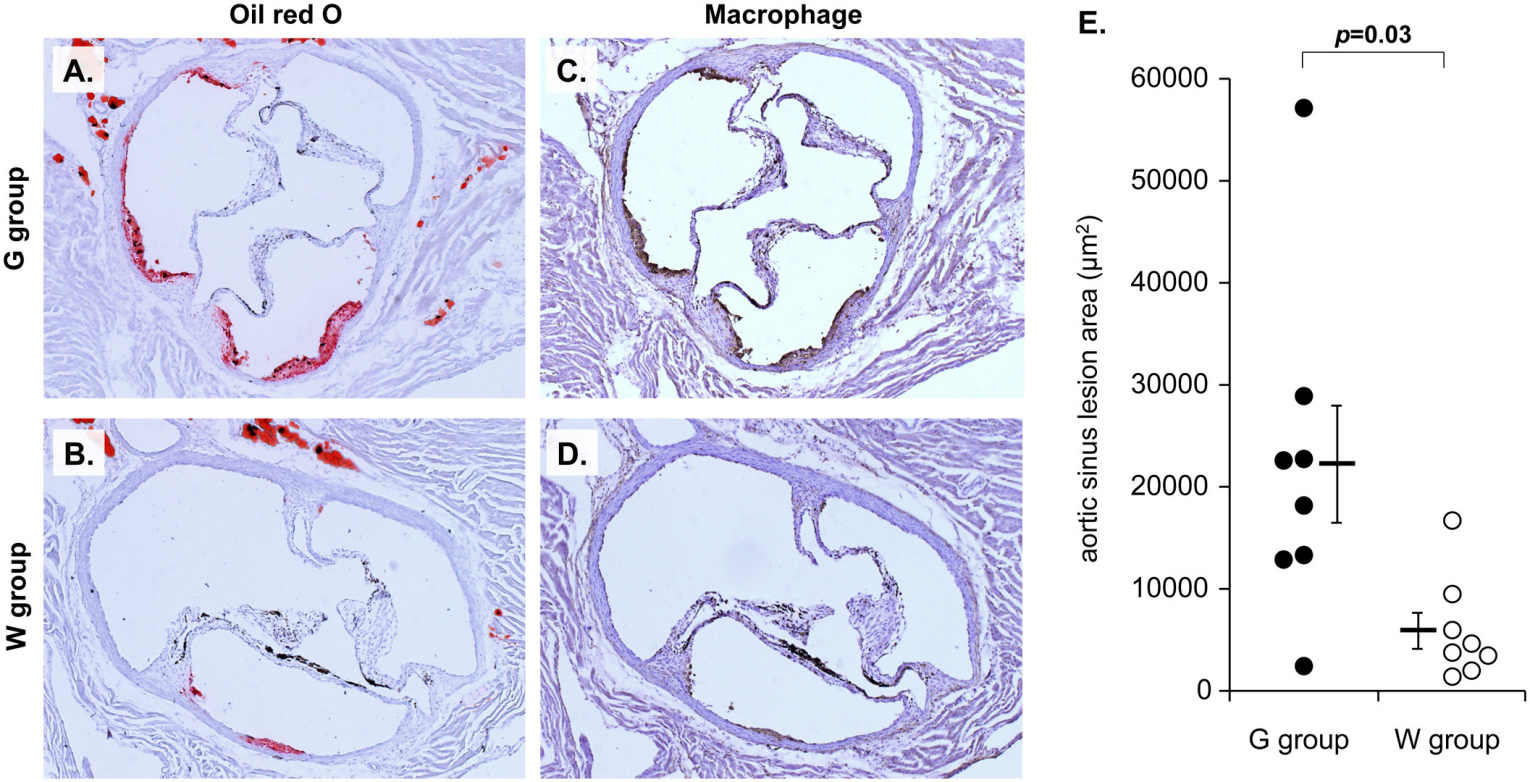
Atherosclerotic lesions in aortic sinus. (A,B) Representative images of oil red O-stained atherosclerotic lesions in aortic sinus. (C,D) Immunohistochemical staining of macrophages (brown) in the cross-sections adjacent to the oil red O-stained images. (E) Quantitative analysis of the lesion area. Each dot indicates mean size of the oil red O-stained lesion area in each mouse. Bars indicate mean ± SEM of 8 mice for each group.

After the 20-week administration period, lipid-laden plaque was observed in the intimal area of the aortic sinus of both groups of mice (Fig 3A and 3B). Atherosclerotic lesion formation was confirmed by the immunohistochemical detection of macrophages (Fig 3C and 3D). The lesion size in G group mice was approximately 4-fold greater than that in W group (Fig 3E).

### Gene expression in thoracic aorta

**Fig 4:**
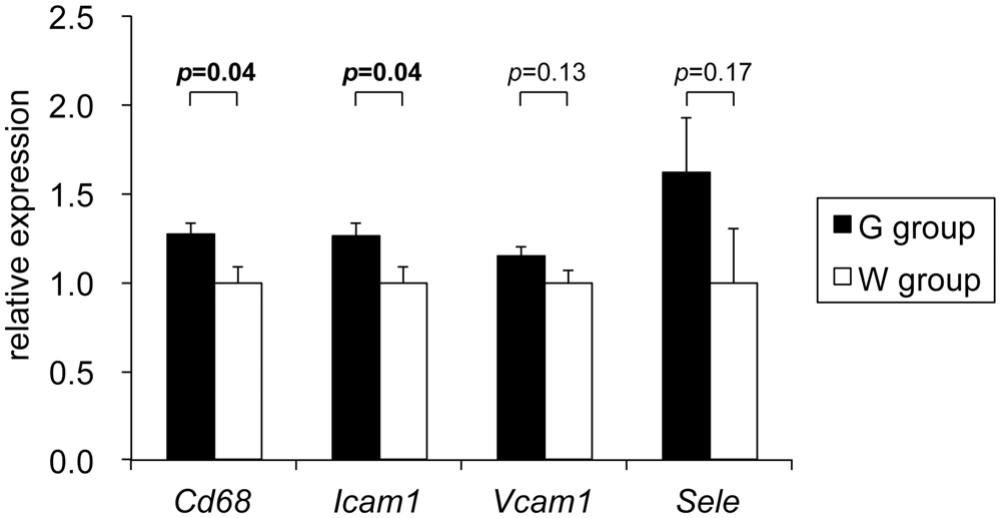
Relative gene expression levels in thoracic aorta. Gene expression levels were normalized to *Actb* (β-actin) in each mouse and expressed as relative values to the mean expression levels in W group. *Icam1*, intercellular adhesion molecule-1; *Vcam1*, vascular cell adhesion molecule-1; *Sele*, E-selectin. Values are expressed as mean ± SEM of 8 mice for each group.

Gene expression levels of a macrophage marker *Cd68* and an adhesion molecule *Icam1* (intercellular adhesion molecule-1) were significantly higher in G group mice than those in W group in the thoracic aorta (Fig 4). Similar trends toward higher gene expression levels in G group mice were also seen for other adhesion molecules *Vcam1* (vascular cell adhesion molecule-1) and *Sele* (E-selectin), though they did not reach statistical significance.

## Discussion

In this study, we clearly demonstrated that repetitive glucose spikes accelerate atherosclerotic lesion formation in wild-type C57BL/6 mice. During the 20-week administration period, G group mice showed acute and transient hyperglycemia just after oral glucose doses. After the administration period, no significant differences were observed in body weight, glucose tolerance, insulin sensitivity, plasma insulin level, and plasma lipid profiles between G and W groups. Taken together, the augmented atherosclerotic lesion formation in G group mice was considered predominantly to be the result of repetitive glucose spikes, with little influence of chronic metabolic disorders such as chronic hyperglycemia, insulin resistance, and dyslipidemia.

A number of observational epidemiological studies have demonstrated an increased cardiovascular risk not only in individuals with diagnosed diabetes but also in those with IGT [2,3]. Furthermore, post-interventional observations of the Diabetes Chronic Complications Trial (DCCT) and the United Kingdom Prospective Diabetes Study (UKPDS) showed that intensive glycemic control early in the course of diabetes could reduce subsequent cardiovascular events [15,16]. These findings imply that glucose spikes in individuals with pre-diabetes or early-stage diabetes may play a crucial role in the pathogenesis of atherosclerosis. In fact, blunting postprandial hyperglycemia (by an α-glucosidase inhibitor acarbose) in subjects with IGT resulted in decreased cardiovascular events in the Study to Prevent Non-Insulin-Dependent Diabetes (STOP-NIDDM) [17].

Besides the epidemiological results, several laboratory studies have addressed the causal role of glucose fluctuations in the pathogenesis of atherosclerosis. In cell culture experiments, for instance, oscillating glucose concentrations have been demonstrated to induce more deleterious pro-atherogenic changes via increased reactive oxygen species production in endothelial cells compared to constant high glucose concentration [18–20]. Transient hyperglycemia-induced oxidative stress and endothelial dysfunction have also been reported in human studies [21–23].

Studies in animal models would be helpful to evaluate pivotal pathways that substantially contribute to the pathogenesis of atherosclerosis *in vivo*. However, most of the existing animal models for investigating the role of hyperglycemia in atherosclerosis are genetically hypercholesterolemic mice (*e*.*g*., apolipoprotein E [apoE] or low-density lipoprotein receptor-deficient mice), in combination with streptozotocin-induced pancreatic β-cell destruction or crossbreeding with genetically obese diabetic mice [24,25]. Since those mice display severe chronic hyperglycemia, they serve as models for overt diabetes rather than for IGT. In addition, severe hypercholesterolemia-induced highly accelerated development of atherosclerosis in these mice often masks the glycemic effect on the atherosclerotic process [26,27]. For example, similar to the present study, Mita et al. [28] previously reported augmented atherosclerotic lesion formation with repetitive maltose administration in apoE-deficient mice; however, the increment in lesion size (~1.5-fold relative to the water-administered control mice) was less pronounced compared to that in the present study (~4-fold). The overwhelming impact of hypercholesterolemia in apoE-deficient mice might make it difficult to accurately assess the glycemic effect on the development of atherosclerosis. Hence, the present approach using wild-type C57BL/6 mice may be more appropriate to clearly demonstrate the atherogenic role of glucose spikes than that using genetically hypercholesterolemic mice.

In contrast to the existing animal models, the simple approach of the present study (*i*.*e*., repetitive oral glucose administration in wild-type mice) successfully induced repetitive glucose spikes and accelerated atherosclerosis without chronic hyperglycemia and overt hypercholesterolemia. In addition, since we used wild-type mice in this study, obvious lipid-laden lesion formation was limited in the aortic sinus, and the size was much less than that in genetically hypercholesterolemic mice. Taken together, the present method would be suitable for investigating the effect of repetitive glucose spikes on the early stages of atherosclerosis. For instance, since the lesion size was evidently different between the 2 groups after the 20-week administration period, biochemical and histological analyses of the causative mechanisms in the aortic tissues are now of great interest. In gene expression analysis, G group mice showed signs of increased macrophage infiltration and elevated cell adhesion molecule expression in the thoracic aorta, where lipid-laden lesions are hardly detectable in wild-type mice, even after long-term atherogenic diet feeding. These results in the thoracic aorta, the adjacent part of the lipid-laden lesion site, would imply the causal atherogenic changes in the arterial wall. A number of studies have demonstrated the role of cell adhesion molecules (including ICAM-1, VCAM-1, and E-selectin) in atherogenesis in knockout mice, although those results have not yet been conclusive [29]. In cell culture experiments, intermittent high glucose was reported to induce ICAM-1, VCAM-1, and E-selectin expression via protein kinase C and mitochondrial superoxide production [19]. Post-glucose load increase in circulating levels of ICAM-1, VCAM-1, and E-selectin was also observed in humans [30]. In addition, increased monocyte adhesion to the thoracic arterial wall was reported in animals experienced blood glucose fluctuations [28, 31]. These pro-atherogenic changes may therefore actually precede and predispose to the development of atherosclerosis.

There are several limitations to this study. First, although repetitive glucose spikes actually accelerated atherosclerotic lesion formation, the *in vivo* molecular mechanisms still remain largely obscure. Further research is needed to investigate the potential causative mechanisms (*e*.*g*., involvement of repetitive transient hyperinsulinemia in response to the glucose spikes, advanced glycation end product-related changes, and protein expression analysis) in arterial tissues. Second, although statistical differences were observed in lesion size in the aortic sinus and some gene expression levels in the thoracic aorta between the 2 groups, large individual variations were seen in the values. A larger sample size study with analyzing biological functions (*e*.*g*., monocyte adhesion, transmigration, and macrophage form cell formation *in vivo*) will therefore be necessary for more convincing evidence. Third, a cholate-containing atherogenic diet (Paigen diet) causes several adverse effects, including cholesterol gallstones and fatty liver, whereas cholate ensures cholesterol absorption and consequent atherosclerotic lesion formation in wild-type mice. Hence, the present study with Pagain diet may not be suitable for evaluating systemic metabolic disorders other than atherosclerosis. Fourth, as mentioned above, atherosclerotic lesions are small and can be formed in the limited area of aortic sinus in wild-type mice. Thus, other methodologies will be required to fully understand the impact of glucose spikes on subsequent severe cardiovascular events such as acute coronary syndromes.

## Conclusions

The present results demonstrate that repetitive glucose spikes can accelerate atherosclerotic lesion formation in mice, with little influence of other metabolic disorders. The present simple method of repetitive glucose administration in wild-type mice with atherogenic diet feeding may serve as a useful approach to address the underlying pathophysiological links between glucose spikes and the development of atherosclerosis in IGT.

## Supporting Information

S1 ARRIVE Checklist (PDF)
